# Network Symmetry and Binocular Rivalry Experiments

**DOI:** 10.1186/2190-8567-4-12

**Published:** 2014-05-07

**Authors:** Casey O Diekman, Martin Golubitsky

**Affiliations:** 1 Department of Mathematical Sciences, New Jersey Institute of Technology, Newark, NJ, 07102, USA; 2 Mathematical Biosciences Institute, The Ohio State University, Columbus, OH, 43210, USA

**Keywords:** Neuronal networks, Rivalry, Coupled cell systems, Symmetry-breaking

## Abstract

Hugh Wilson has proposed a class of models that treat higher-level decision making as a competition between patterns coded as levels of a set of attributes in an appropriately defined network (Cortical Mechanisms of Vision, pp. 399–417, 2009; The Constitution of Visual Consciousness: Lessons from Binocular Rivalry, pp. 281–304, 2013). In this paper, we propose that symmetry-breaking Hopf bifurcation from fusion states in suitably modified Wilson networks, which we call *rivalry networks*, can be used in an algorithmic way to explain the surprising percepts that have been observed in a number of binocular rivalry experiments. These rivalry networks modify and extend Wilson networks by permitting different kinds of attributes and different types of coupling. We apply this algorithm to psychophysics experiments discussed by Kovács et al. (Proc. Natl. Acad. Sci. USA 93:15508–15511, 1996), Shevell and Hong (Vis. Neurosci. 23:561–566, 2006; Vis. Neurosci. 25:355–360, 2008), and Suzuki and Grabowecky (Neuron 36:143–157, 2002). We also analyze an experiment with four colored dots (a simplified version of a 24-dot experiment performed by Kovács), and a three-dot analog of the four-dot experiment. Our algorithm predicts surprising differences between the three- and four-dot experiments.

## 1 Introduction

In standard binocular rivalry experiments, the left and right eyes of the subject are presented dissimilar images, and the subject’s perception alternates between the two presented images [1]. Perceptual alternation has often been modeled as competition between two units, each representing a percept, with reciprocal inhibition between the units (cf. [2-6]). The point of those models is to analyze the dynamics of alternation assuming a set of percepts, rather than predicting the form of the percepts. In contrast, the goal of our analysis is to predict the form of percepts, and not the precise dynamics of alternation. To do this, we use symmetry-breaking and an extension of a class of network models proposed by Wilson [7], which we call *rivalry networks*. Our analysis is model-independent; that is, we concentrate on the network architecture and not on specific equations associated with the networks.

Wilson networks generalize binocular rivalry models to multiple competing patterns [7,8]. Originally intended as a model of deliberative decision making, these networks embody aspects of conscious brain processes that have been discussed previously by many other researchers, such as Tononi and Edelman [9], Dehaene et al. [10,11], and Crick and Koch [12]. A Wilson network consists of a set of attributes relevant to a given decision, with reciprocal inhibition among the nodes associated with each attribute. A pattern consists of one node from each attribute, with excitatory coupling among nodes in a learned pattern. In [13], we showed that Wilson networks could be constructed to represent the binocular rivalry experiments of Kovács et al. [14], and that certain states of these Wilson networks corresponded to the unexpected percepts observed in [14]. This result suggested that Wilson networks can help classify and predict percepts for binocular rivalry experiments. In this paper, we expand and modify the ideas in [13] to demonstrate that Wilson-type models can be constructed in an algorithmic way for several binocular rivalry experiments in the literature, and that these models seem to explain the surprising percepts observed in these experiments. Using these models we also predict percepts for several binocular rivalry experiments that have yet to be performed. 

Our algorithm has three parts. First, we generalize Wilson networks by introducing different types of nodes (attributes) and different types of excitatory couplings (based on *features*). An example of attributes of the same type is the color of discs at different geometric locations. An example of different attribute types is the color of a disc and the direction of a grating pattern in that disc. Features are properties of pairs of nodes, such as the geometric distance between two discs. We use features (which are based on Hebbian learning) to determine whether coupling between pairs of nodes are assumed to be the same or not. It is the systematic use of features, and hence of multiple types of excitatory coupling, that makes our symmetry-breaking analysis possible. Second, we use network symmetries to identify the *maximal fusion states* (see Definitions 1 and 2) of the network. Finally, we classify the types of *fusion-breaking* Hopf bifurcation from maximal fusion states; that is, Hopf bifurcations that lead to non-fusion states. The resulting periodic states lead to alternation in network patterns that we interpret as predictions of the alternations in perception a subject would be likely to experience. We claim that for Hopf bifurcation to lead to alternation between percepts, the bifurcation must take place from a fusion state. Furthermore, the expectation that fusion states exist depends on network architecture (specifically on network symmetry). This point is discussed in Sect. 2.1.

We demonstrate this procedure on the *monkey-text* experiments of Kovács et al. [14], the *geometric rivalry* stimuli experiments in Suzuki and Grabowecky [15], and the *color misbinding* experiments of Shevell and Hong [16,17]. The types of periodic solutions that come from fusion-breaking bifurcations in the rivalry networks we construct are consistent with the surprising percepts reported in these experiments. Moreover, we make testable predictions about the states that we expect to be perceived in *colored-dot* experiments that have yet to be performed. We predict qualitatively different sets of percepts for three colored-dot and four colored-dot stimuli. In particular, for the four-dot experiments, the predictions from our theory include simple alternation between the presented stimuli, as one would intuitively expect to occur. However, for certain three-dot experiments, our theory predicts that such alternation should not occur generically (see Sect. 6.3).

### 1.1 Wilson Networks

**Fig. 1:**
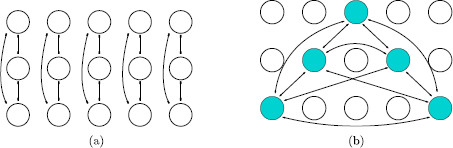
Architecture for a Wilson network. **a** Inhibitory connections between nodes in an attribute column are denoted by *lines with rectangular ends*. **b** Excitatory connections in a learned pattern are denoted by *lines with arrowheads*

Wilson networks, as codified in [18], are made up of attribute columns, with each column containing a set of nodes corresponding to levels of that attribute. *Patterns* correspond to the choice of one level in each attribute column. Wilson assumes that the nodes in a column are all-to-all connected by inhibitory couplings and that the network has a set of learned patterns. The nodes in a *learned* pattern are assumed, based on Hebbian learning, to be all-to-all coupled by excitatory connections. See Fig. 1 for an example of a five-attribute three-level Wilson network with one learned pattern. 

Wilson assumes that these networks can have multiple learned patterns and that rivalry is represented by solutions that alternate between learned patterns. In [13] we observed that network periodic solutions can exhibit patterns that were not learned, which we call *derived*. We proposed that the unexpected percepts in many binocular rivalry experiments exist because the dynamics of associated Wilson networks lead naturally to particular derived patterns. As noted, in this paper we generalize the definition of Wilson networks to *rivalry networks*, which include detail that was left out of the discussion in [13], and which is needed to systematically determine networks for binocular rivalry experiments. In doing so we use the theory of coupled cell networks developed in [19,20]. 

Wilson networks [7,8] consist of a rectangular grid of nodes with *n* attribute columns, *m* levels in each column, and *ℓ* learned patterns. There is some leeway in choosing differential equations associated to a given Wilson network. Wilson [7] and others assume that the nodes are neurons or groups of neurons and that the important information is captured by the firing rate of the neurons. In these models each node (i,j) has a state space $x_{ij}=(x_{ij}^{E},x_{ij}^{H})$, where $x_{ij}^{E}$ is an activity variable (representing firing rate) and $x_{ij}^{H}$ is a fatigue variable. Coupling between nodes is given through a gain function . Specifically, for level *i* in attribute *j* we have the system

(1) $$\begin{array}{rl} \varepsilon {\dot{x}}_{ij}^{E} & =-x_{ij}^{E}+G\left(I_{ij}+w\sum_{pq\to ij}x_{pq}^{E}-\beta \sum_{rj⊣ij}x_{rj}^{E}-gx_{ij}^{H}\right),\\ {\dot{x}}_{ij}^{H} & =x_{ij}^{E}-x_{ij}^{H}, \end{array}$$

where → indicates an excitatory learned pattern connection and ⊣ indicates an inhibitory connection. The parameters are: reciprocal learned pattern excitation between nodes w>0, reciprocal inhibition between nodes β>0, the external signal strength $I_{ij}\geq 0$ to nodes, the strength of reduction of the activity variable by the fatigue variable g>0, and the ratio of time scales ε<1 on which ${*}^{E}$ and ${*}^{H}$ evolve. The gain function  is usually assumed to be nonnegative and nondecreasing, and is often a sigmoid.

In this paper we assume only that each node (i,j) has an *activity* variable $A_{ij}\in R$. The activity variable is just the coordinate $x_{ij}^{E}$ in (1), but in general it might be a function of the state space variables $A_{ij}=A_{j}(x_{ij})$. As indicated, this function is assumed to be the same for each level of an attribute column. The choice of level *i* in an attribute column *j* occurs when $A_{ij}>A_{kj}$ for all k≠i. A percept is assumed to be *dominant* when the activity variable of each node associated with the pattern is a unique maximum within its attribute column.

#### 1.1.1 Fusion States

**Definition 1** A *fusion state* is one where the maximum of the $A_{ij}$ in some column occurs simultaneously in more than one level.

For general systems of differential equations we would not expect fusion states to be important. Specifically, suppose a system had a fusion equilibrium. Then one can always perturb the differential equation slightly so that the fusion equilibrium moves to a state where the maximum value of $A_{ij}$ in each column is unique; that is, fusion states are destroyed by small perturbations. However, network architecture, usually through its symmetries, can force the existence of structurally stable fusion states. More precisely, let *Σ* be a set of network symmetries. Then the *fixed-point subspace* of *Σ*,

Fix(Σ)={X:σX=X for all σ∈Σ},

is well known [20,21] to be a flow-invariant subspace of the state space of the network admissible differential equations. So it is not surprising to find solutions (equilibria or periodic solutions) in Fix(Σ), since those fusion solutions cannot be gotten rid of by perturbation. If one of the symmetries maps one node in an attribute column to another node in that same column, then the corresponding fixed-point subspace can have fusion states that cannot be destroyed by perturbation of the differential equation.

**Definition 2** Let *Γ* be the group of network symmetries for a fixed network. We call states in Fix(Γ)*maximally fused* states.

Those Hopf bifurcations that can lead to a non-fused periodic solution from a maximally fused equilibrium are called *fusion-breaking* Hopf bifurcations. Since such bifurcations usually lead to periodic solutions with nonzero phase shifts between the previously fused nodes, these bifurcations lead to rivalrous solutions.

Note that modeling alternation between percepts (patterns) in networks of differential equations requires finding periodic solutions that alternate periods of dominance; that is, for part of the trajectory one set of nodes has maximum activity and during another part of the trajectory a different set of nodes has maximum activity. Thus, during the trajectory there must be times when the activity of multiple nodes are equal. A small amplitude periodic solution obtained via Hopf bifurcation can have this property only if the equilibrium from which the bifurcation takes place is a fusion equilibrium. We assume, as is standard in bifurcation theory in the presence of symmetry, that the states that are most likely to be observed are spawned by bifurcation from a maximally fused (or symmetric) state.

### 1.2 The Structure of the Paper

In this paper we discuss several binocular rivalry experiments. For each experiment we formulate a rivalry network, compute the fusion-breaking Hopf bifurcations, and discuss the derived percepts. Specifically, we discuss rivalry models for the conventional and scrambled *monkey*-*text* experiments of Kovács et al. [14] in Sect. 2; the geometric shapes of Suzuki and Grabowecky [15] in Sect. 3; and the color misbinding experiments of Shevell et al. [16,17] in Sect. 4. The Shevell experiments require having rivalry networks with two types of nodes. In Sects. 5 and 6, we analyze experiments with three or four colored dots, which are analogs of the 24 colored dot experiments in Kovács et al. [14]. Summaries of our predictions are given in Sect. 7. This analysis requires the use of features; that is, of several types (or strengths) of excitatory coupling.

We give a formal definition of rivalry networks in Sect. 8 and summarize our approach to rivalry-driven percepts in Sect. 9.

## 2 *Monkey*-*Text* Experiments

**Fig. 2:**
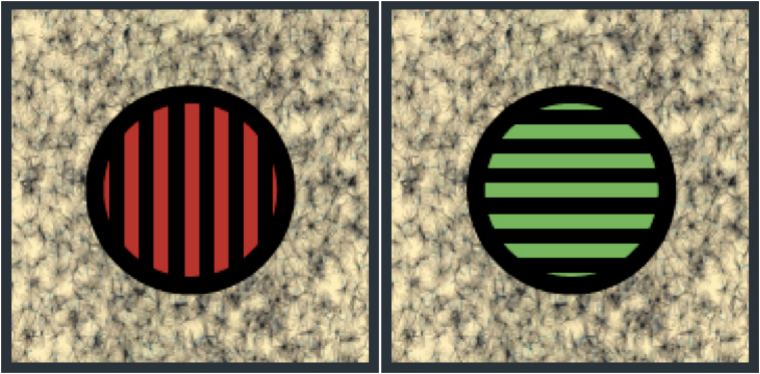
A standard rivalry experiment from Blake and Logothetis [1]

In a standard rivalry experiment, the subject is shown two images (such as those in Fig. 2) simultaneously; one image is shown only to the left eye, while the other image is shown only to the right eye. The subject is then asked what he or she observes. Typically the subject perceives the two images alternating in time. The perceived images are called *percepts*. 

### 2.1 A Symmetry Analysis of Standard Rivalry

**Fig. 3:**
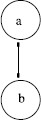
Network for standard rivalry. Symmetry group is $\Gamma =Z_{2}$

Much of the modeling of standard rivalry assumes that there are two units: one for each of the percepts. Moreover, the two units have reciprocal inhibitory coupling, as in Fig. 3. The model equations are usually assumed to have the form

(2) $$\begin{array}{rl} \dot{a} & =f(a,b),\\ \dot{b} & =f(b,a). \end{array}$$

Suppose that (2) has a winner-take-all state; that is, an equilibrium where a≠b. Generically, we may assume that either $a^{E}>b^{E}$ or $a^{E}<b^{E}$. Let us assume the former so that the percept *a* is what is perceived. Then Hopf bifurcation from this equilibrium will lead to a small amplitude periodic state where $a^{E}(t)>b^{E}(t)$ for all *t*. Thus, the perceived state will still be the constant perception of percept *a*; there will not be any alternation. Indeed, the only way that Hopf bifurcation can lead to percept alternation is if that Hopf bifurcation is from a fusion state where a=b. It is then fair to ask why in a given model (a specific choice of *f* in (2)) it would be reasonable to assume that there is a fusion equilibrium. The reason is symmetry. 

Equations (2) have *ρ* symmetry, where ρ(a,b)=(b,a) permutes the two units. Observe that

Fix(ρ)≡{(a,b):ρ(a,b)=(a,b)}={(a,a)};

that is, the fixed-point subspace of *ρ* consists of fusion states (those states for which b=a). A standard fact from dynamical systems with symmetries is that fixed-point subspaces are flow-invariant (cf. [21]), and this fact can be checked directly for (2). It is therefore a reasonable hypothesis that a model like (2) has a fusion equilibrium.

Note that *ρ* has order two (applying *ρ* twice leads to the identity). We denote the group of symmetries consisting of *ρ* and the identity by $Z_{2}$ or sometimes by $Z_{2}(\rho )$. Due to the $Z_{2}$ symmetry, there are two types of Hopf bifurcation from a fusion state in (2); one leads to periodic fusion states (a(t),a(t)), and the other to *T*-periodic solutions of the form

(3) $$b(t)=a\left(t+\frac{T}{2}\right).$$

Such periodic solutions will have the two percepts alternating periods of dominance and are thought to model the rivalry state. Observe that the Jacobian of (2) at a fusion state has the form

$$J=(\begin{array}{cc} \alpha & \beta \\ \beta & \alpha \end{array})$$

and that *J* has invariant subspaces

$$V^{+}=(\begin{array}{c} a\\ a \end{array})\;\text{and}\;V^{-}=(\begin{array}{c} a\\ -a \end{array}).$$

Moreover, *J* acts as multiplication by α+β on $V^{+}$ and as multiplication by α−β on $V^{-}$. Thus, the eigenvalues of *J* are the eigenvalues of α+β and the eigenvalues of α−β. If the critical eigenvalues for *J* are associated to α+β, then Hopf bifurcation will lead to the fusion states (a(t),a(t)); if the critical eigenvalues for *J* are associated to α−β, then Hopf bifurcation will lead to the non-fusion states (3).

We find it convenient to use the notation

$$a_{\theta }(t)=a(t+\theta T).$$

Then the two types of periodic solution that can be obtained by Hopf bifurcation from a fusion state have the form

$$\left(\begin{array}{c} a_{0}\\ a_{0} \end{array}\right)$$

and

(4) $$(\begin{array}{c} a_{0}\\ a_{1/2} \end{array}).$$

Thus, there is only one fusion-breaking Hopf bifurcation and that bifurcation leads to alternation between the two learned images.

#### 2.1.1 A Short Summary of Hopf Bifurcation in the Presence of Symmetry

Our discussion of Hopf bifurcation in the presence of $Z_{2}$ symmetry is actually quite general. Observe that the symmetry *ρ* acts trivially on $V^{+}$ and as multiplication by −1 on $V^{-}$. These two subspaces correspond to the trivial one-dimensional irreducible representation of $Z_{2}$ and the nontrivial one-dimensional representation of $Z_{2}$.

For a general group *Γ* there is a type of Hopf bifurcation that corresponds to each type of irreducible representation *V*. The general theory of Hopf bifurcation in the presence of symmetry as well as the results of these bifurcations for a number of groups *Γ* and their irreducible representations can be found in [21,22]. The examples worked out in these references include all of the groups that appear in this paper. The symmetry groups that will appear in this paper are $Z_{2}$, $D_{n}$, and $D_{n}⊕Z_{2}$, where $D_{n}$ is the symmetry group of the 2*n* symmetries of a regular *n*-gon (a rectangle when n=2, an equilateral triangle when n=3, and a square when n=4). Note that the symmetries of a rectangle are generated by two reflections; that is, $D_{2}=Z_{2}⊕Z_{2}$. All of these groups will appear as permutation symmetries of a network. For example, $D_{3}$ will appear as the permutation group of a ring of three identical nodes.

Finally, if a symmetry γ∈Γ acts trivially on *V*, then all solutions x(t) corresponding to the associated Hopf bifurcation will satisfy γx(t)=x(t). Moreover, if a symmetry γ∈Γ acts as multiplication by −1 on *V*, then all *T*-periodic solutions x(t) corresponding to the associated Hopf bifurcation will satisfy γx(t)=x(t+T/2). If *γ* permutes nodes in the same attribute column, then the first result leads directly to fusion states and the corresponding Hopf bifurcation is not fusion-breaking.

### 2.2 Conventional *Monkey*-*Text* Experiment

**Fig. 4:**
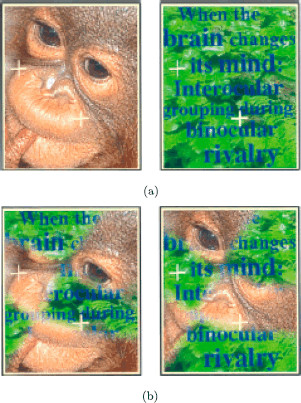
From [14]. **a** Learned images in *monkey*-*text* rivalry experiment. **b** Learned images in scrambled *monkey*-*text* experiment

Kovács et al. [14] describe two rivalry experiments based on the images of a monkey and a jungle scene with text shown in Fig. 4a. In the *conventional* experiment, the subject is presented these two images. In the *scrambled* experiment, the subject is presented images that have been reassembled from subdivisions of the original images (see Fig. 4b). These experiments were discussed in [13], where the surprising results of the scrambled experiment were shown to correspond to derived patterns in a Wilson network. In this section we give a different derivation of the Wilson network, one that is algorithmic and generalizes to other experiments. 

The conventional *monkey*-*text* experiment is a standard rivalry experiment. In this experiment there is one attribute (the image) and it has two levels (*monkey* and *text*). There is no excitatory coupling in the network and the network is the one shown in Fig. 3. As expected, the subjects in this experiment report alternation between the *monkey* image and the *text* image.

### 2.3 Scrambled *Monkey*-*Text* Experiment

**Fig. 5:**
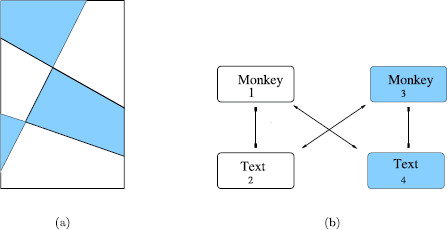
**a** Distinct areas in scrambled *monkey*-*text* experiment. **b** Two-attribute two-pattern Wilson network for scrambled *monkey*-*text* experiment with reciprocal inhibition in attribute columns and reciprocal excitation in learned patterns. The symmetry group is $\Gamma =D_{2}$

The scrambled *monkey*-*text* images are formed by cutting the *monkey* and the *text* images into six pieces each and then assembling new images by alternating the cut pieces from the two original images. The two scrambled images are formed as follows. The first learned image has *monkey* in the blue areas of the rectangle in Fig. 5a and *text* in the white areas and the second learned image has *text* in the blue areas and *monkey* in the white areas. 

We form a Wilson network with two attributes (the image type in the blue area and the image type in the white area). Each attribute has two levels: *monkey* and *text*. We assume that the levels all have the same type and that there is just one type of excitatory coupling. These assumptions lead to the Wilson network in Fig. 5b, whose nodes are labeled 1, 2, 3, 4 and whose corresponding state space is $\left(x_{1},x_{2},x_{3},x_{4}\right)$ where $x_{j}\in R^{k}$. This network has symmetry group $\Gamma =D_{2}(\rho ,\kappa )$ where the permutation *ρ* swaps rows and the permutation *κ* swaps columns. Specifically,

$$\begin{array}{rcl} \rho & = & (1\;2)(3\;4),\\ \kappa & = & (1\;3)(2\;4). \end{array}$$

Since $D_{2}$ acts transitively on the nodes, the maximal fusion states satisfy $x_{1}=x_{2}=x_{3}=x_{4}$.

Symmetry permits four types of symmetry-breaking Hopf bifurcations from a maximal fusion state, and their associated center subspaces have the forms in (5):

(5) $$\begin{array}{rclll} V^{++} & = & \left\{(a,a,a,a)\right\} & \rho =1=\kappa & \text{fusion},\\ V^{+-} & = & \left\{(a,a,-a,-a)\right\} & \rho =1,\kappa =-1 & \text{fusion},\\ V^{-+} & = & \left\{(a,-a,a,-a)\right\} & \rho =-1,\kappa =1 & \text{rivalry: derived},\\ V^{--} & = & \left\{(a,-a,-a,a)\right\} & \rho =-1=\kappa & \text{rivalry: learned.} \end{array}$$

Those isotypic components for which *ρ* acts trivially on the center subspace lead to periodic fusion states. There are two bifurcations where *ρ* acts nontrivially: $V^{-+}$ where *κ* acts trivially (which leads to rivalry between the learned scrambled states) and $V^{--}$ where *κ* acts nontrivially (which leads to rivalry between the unscrambled states). The periodic solutions corresponding to these bifurcations have the form

$$(\begin{array}{cc} a_{0} & a_{0}\\ a_{1/2} & a_{1/2} \end{array})\;\text{and}\;(\begin{array}{cc} a_{0} & a_{1/2}\\ a_{1/2} & a_{0} \end{array}).$$

Our algorithm indicates that these two types of periods of alternation are possible. One type is a period of alternation between the scrambled images and the other type is a period of alternation between the reconstructed conventional images.

### 2.4 Comparison with the Results in [13]

In a previous paper [13], we showed that the scrambled experiment could be understood using the network in Fig. 5b. However, when one uses the rate equations (1), rivalry solutions between the derived patterns can never be stable at bifurcation. We addressed this point by adding a second form of excitatory coupling, which we called lateral coupling, to the network. We no longer believe that stability is a crucial issue, since we do not know what the equations that correspond to a rivalry network should be. Here, we focus on the existence of patterns of alternation and therefore have no need to introduce lateral coupling. These results depend only on network structure and not on specific model equations; that is, these results are *model-independent*.

## 3 Rivalry Between Geometric Shapes

**Fig. 6:**
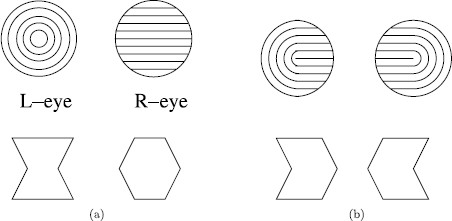
Percepts for Suzuki and Grabowecky [15]

Suzuki and Grabowecky [15] report results of four different rivalry experiments, all of which have a common description. The left eye and right eye images are each split into a left-half image and a right-half image. The surprising percepts are ones that group the left-half of the left eye image with the right-half of the right eye image and the right-half of the left eye image with the left-half of the right eye image. The images presented in two of the four experiments are shown in Fig. 6a, and the corresponding surprising percepts are shown in Fig. 6b. 

It is not clear why the brain divides the images into left halves and right halves, yet that is what appears to be happening. Note that the Wilson network for the Suzuki and Grabowecky [15] experiments is isomorphic to the Wilson network for scrambled *monkey*-*text* experiment in Fig. 5b. The network has two attributes (left-half image and right-half image) and two levels (the geometric shape on the appropriate side).

There are two questions that arise when modeling a rivalry experiment: what are the states (i.e. the form of the percepts), and what is the dynamics of switching among percepts? In this paper we are focused on the former, which depends on network structure and not on specific model equations. However, Suzuki and Grabowecky [15] described an interesting property of rivalry dynamics that we would like to comment on. In their experiments, subjects exhibited a bias in the transitions among states called *perceptual trapping*. For example, consider the hourglass–diamond–chevrons set shown in the bottom row of Fig. 6. Whichever pair of images the subjects were shown (either the hourglass–diamond pair of Fig. 6a, or the chevron pair of Fig. 6b), the perceived shape alternated between the hourglass and the diamond and between the two chevrons more often than would be expected if the transitions among the four perceived images were random. Transitions between these pairs, i.e. from an hourglass or a diamond to a chevron or vice versa, occurred less often. Although this trapping phenomenon is not captured explicitly by our model, it is consistent with our observation that there are two different types of symmetry-breaking Hopf bifurcation that lead to rivalry in two-attribute two-level networks such as Fig. 5b. Alternation between the rivalry associated with each of these bifurcations could occur if stochastic effects were included in the model. If the noise-induced alternations occurred on a slower time scale than the oscillations arising from Hopf bifurcation, then overall the dynamics would resemble perceptual trapping.

## 4 Color Misbinding

Shevell et al. [16,17] show that rivalry between images that have static gratings of different colors can lead to *color misbinding*: percepts with gratings comprised of one color from each of the learned patterns. This experiment enables us to explore the use of rivalry networks with more than one attribute type.

### 4.1 Two Grating Directions

**Fig. 7:**
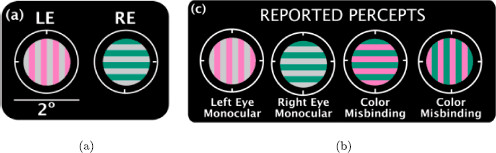
Visual stimuli (**a**) and reported percepts (**b**) in Shevell et al. [17]

In the rivalry experiment of Shevell et al. [17], the subjects are presented the images in Fig. 7a and report the percepts shown in Fig. 7b. 

We propose a rivalry network with three attributes: one grating direction and two colors (one in the top/left and one in the bottom/right). The grating direction has two levels (horizontal and vertical) and the color attributes have three levels (pink, gray, green). We assume that there is one type of excitatory coupling from grating direction to color and another from color to color.

The network symmetry group is $\Gamma =D_{2}(\tau ,\rho )$ where *ρ* swaps appropriate rows in each column and where *τ* swaps nodes in the two color attribute columns. Specifically

$$\begin{array}{rcl} \tau & = & (4\;6)(5\;7)(3\;8),\\ \rho & = & (1\;2)(4\;5)(6\;7),\\ \rho \tau & = & (1\;2)(3\;8)(4\;7)(5\;6). \end{array}$$

**Fig. 8:**
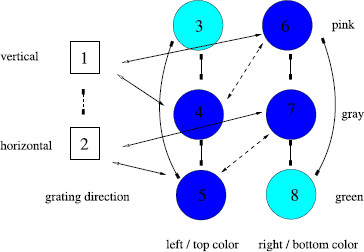
Rivalry network for Shevell et al. [17] experiment. This network has two types of nodes (corresponding to grating direction and color), two types of inhibitory coupling (one for each kind of node), and three types of excitatory coupling (one connecting two color nodes, one connecting a color node to a grating node, and one connecting a grating node to a color node)

The maximal fusion states are indicated by color in Fig. 8. They have nodes 1 and 2 equal; nodes 3 and 8 equal; nodes 4, 5, 6 and 7 equal, as in

$$(\begin{array}{ccc} & b & c\\ a & & \\ & c & c\\ a & & \\ & c & b \end{array}).$$

There are four one-dimensional irreducible representations of $D_{2}$. If either *ρ* or *ρτ* acts trivially on the representation, then $x_{1}=x_{2}$ and the corresponding percepts exhibit fusion in the gratings. Since we are looking for fusion-breaking solutions, we can focus on the one irreducible representation where *ρ* and *ρτ* act as minus the identity and *τ* acts trivially. The corresponding isotypic component has the form

$$(\begin{array}{ccc} & 0 & y\\ x & & \\ & y & -y\\ -x & & \\ & -y & 0 \end{array}).$$

Note that since *τ* implies that $x_{3}=x_{8}$ and *ρτ* implies that $x_{3}=-x_{8}$, it follows that $x_{3}=x_{8}=0$ for states in the isotopic component. Hopf bifurcation corresponding to this representation leads to periodic solutions of the type

(6) $$X(t)=(\begin{array}{ccc} & u_{0} & y_{0}\\ x_{0} & & \\ & y_{0} & y_{1/2}\\ x_{1/2} & & \\ & y_{1/2} & u_{0} \end{array}),$$

where u(t) has twice the frequency of x(t) and y(t) since *τ* implies $u_{1/2}=u_{0}$. If |y|>|u| (that is, bifurcation is from a fusion state where c>b), then the percepts will alternate between pink/gray and gray/green; that is alternation between learned images. In fact, the alternation is predicted to be somewhat more complicated; the moments in time when horizontal and vertical gratings alternate are different from the moments in time when the colors alternate. Shevell et al. [17] report the different percepts but not the order in which the percepts alternate. 

On the other hand, if |y|<|u| (that is, bifurcation is from a fusion state b>c), then the percept will be green/pink, which corresponds to color misbinding. Here, there is no alternation of colors, but there is still alternation in grating direction. Thus, the surprising color misbinding in [17] seems also to be a product of the rivalry network structure. 

### 4.2 One Grating Direction

**Fig. 9:**
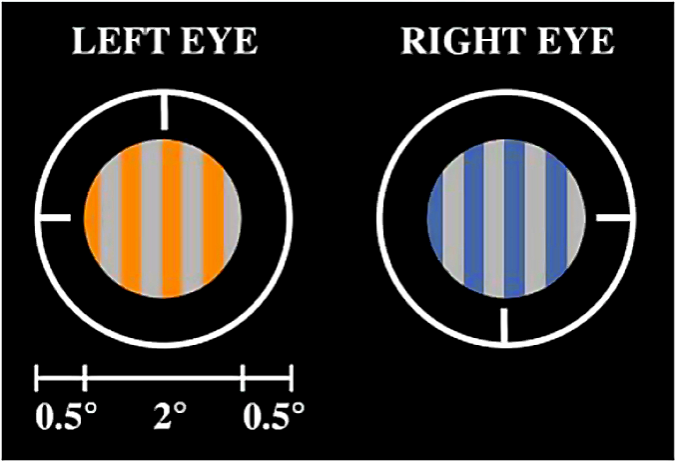
Hong and Shevell [16] stimuli

In the rivalry experiment of Hong and Shevell [16], the two stimuli presented to the two eyes are the ones in Fig. 9. The surprising percept is a grating colored orange and blue. 

**Fig. 10:**
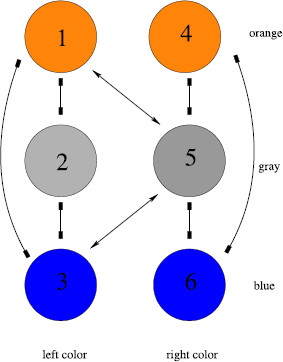
Network for Hong and Shevell [16] experiment

The rivalry network that we propose for the Hong–Shevell experiment has two attributes (left color and right color) and each attribute has three levels (orange, gray, and blue). There is one type of excitatory coupling. See Fig. 10. 

The symmetry group of this network is $\Gamma =D_{2}$ that is generated by the transpositions ρ=(13) and τ=(46). The maximal fusion states have nodes 1 and 3 equal and nodes 4 and 6 equal. There are four types of Hopf bifurcation of $D_{2}$. However, if either *ρ* or *τ* acts trivially then Hopf bifurcation leads to a fusion state. It follows that there is only one relevant Hopf bifurcation from fusion and that bifurcation leads to states of the form

$$\left(\begin{array}{cc} a_{0} & b_{0}\\ c_{0} & d_{0}\\ a_{1/2} & b_{1/2} \end{array}\right)$$

where $c_{0}=c_{1/2}$ and $d_{0}=d_{1/2}$. There are four possible percept alternations. 

• $c_{0}>a_{0}$, $d_{0}>b_{0}$: the percept is an all gray image.

• $c_{0}>a_{0}$, $d_{0}<b_{0}$: the percept alternates gray-orange and gray-blue.

• $c_{0}<a_{0}$, $d_{0}>b_{0}$: the percept alternates orange-gray and blue-gray, which is just alternation between the original stimuli.

• $c_{0}<a_{0}$, $d_{0}<b_{0}$: the percept alternates orange, orange-blue, blue, and blue-orange.

## 5 Four Colored-Dot Experiments

**Fig. 11:**
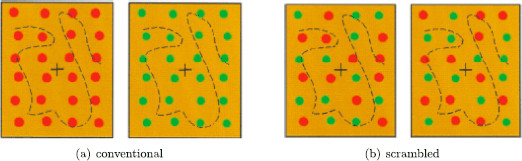
Learned images in *colored-dot* experiments of [14]

The images used in the colored-dot experiments of Kovács et al. [14] are shown in Fig. 11. In the conventional experiment, the image with all red dots is shown to one eye and the image with all green dots is shown to the other eye. In the scrambled experiment, the images shown to each eye contain a mixture of green and red dots as in Fig. 11b. The scrambled dots experiment leads to rivalry between the scrambled images and to rivalry between single color dot percepts, as might be expected from the scrambled *monkey*-*text* experiment. However, the conventional dots experiment leads to another unexpected result: rivalry between mixed-color percepts (such as the images in Fig. 11b), in addition to rivalry between the single colored dot images (Fig. 11a). 

In their review article, Tong et al. [23] suggest focusing on the four centrally located dots in these images and describe the Kovács et al. experiments in these terms. We discussed these experiments in [13], where we used a notion of lateral coupling to propose Wilson-type networks that could help explain the surprising outcomes. Here we propose a different mechanism for deriving rivalry networks for the conventional and scrambled dot experiments, one based on features that lead to different types of excitatory couplings within learned patterns. This approach creates networks that are different from the ones proposed in [13] (though they have the same symmetries). It has the advantage of being consistent with Hebbian learning and with all of the other examples discussed in this paper. We return to these points below. These modeling results can be thought of as predictions, since to our knowledge these experiments have not been performed. 

**Fig. 12:**
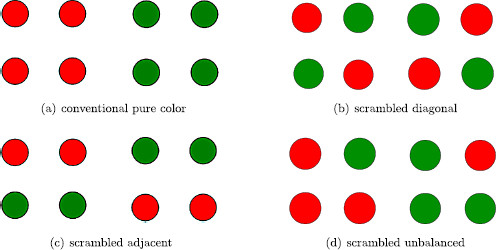
Learned images for two-color *four-dot* experiments

We note that the suggestion in [23] to focus on the four central dots is somewhat arbitrary. Other choices of a square of four dots lead to different configurations and different rivalry networks. Indeed, up to symmetry, there are four different types of four-dot experiments, all of which can be found within the Kovács et al. experiments: *conventional pure color*, *scrambled diagonal*, *scrambled adjacent*, and *scrambled unbalanced*. See Fig. 12 where the left eye and right eye images for each of the four different experiments are shown. 

### 5.1 Four-Dot Networks and Their Symmetries

**Fig. 13:**
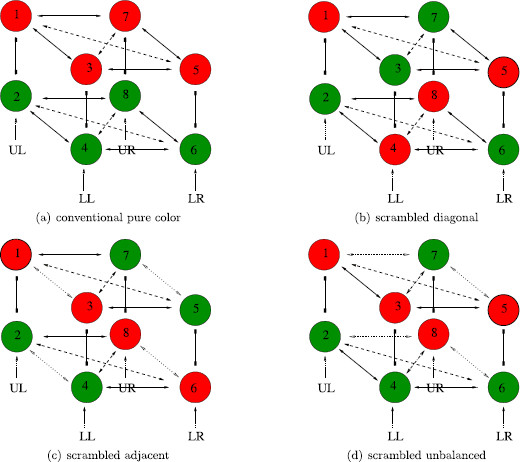
Rivalry networks for two-color *four-dot* experiments. *Solid lines with arrowheads* indicate excitatory coupling between *adjacent dots*, and *dashed lines with arrowheads* indicate excitatory coupling between *diagonal dots*

We assume that the four-dot experiments each have four attributes: the color of the dots at the four geometric positions: upper left (UL), lower left (LL), lower right (LR), upper right (UR). Each attribute has two levels: red and green. Each of the four experiments leads to a different rivalry network, though the first two networks are isomorphic and have the same group of symmetries. See Fig. 13. We show that the expected percepts in the conventional and scrambled diagonal networks are the same, which is consistent with the Kovaćs et al. 24-dot experiments; whereas we expect percepts for the other two experiments to be different. We discuss each network in turn. 

*Conventional pure color* ($D_{4}\times Z_{2}(\rho )$*symmetry*): The two learned patterns consisting of all red and all green dots, respectively, are shown in Fig. 12a. The conventional four-dot network has two types (or strengths) of excitatory coupling, one for adjacent dots and one for diagonal dots. These coupling types are indicated in Fig. 13a by solid and dashed lines with arrows. This is an example of a *feature*; couplings are the same only if the dots are equidistant. See Definition 4(d).

The conventional network has symmetry group $D_{4}\times Z_{2}(\rho )$, where the symmetry group of a square $D_{4}$ permutes the attribute columns and the permutation

ρ=(12)(34)(56)(78)

swaps the upper and lower rows. We represent a point in phase space by the 2×4 matrix

(7) $$(\begin{array}{cccc} x_{1} & x_{3} & x_{5} & x_{7}\\ x_{2} & x_{4} & x_{6} & x_{8} \end{array}),$$

where $x_{j}\in R^{k}$. Since the symmetry group acts transitively, the maximal fusion states have all nodes equal:

(8) $$(\begin{array}{cccc} x & x & x & x\\ x & x & x & x \end{array}).$$

Note that if we assume a standard rate equation model (1) for this eight-node network, then the equations corresponding to a single node *j* will be a two-dimensional system of equations with state variables $x_{j}^{E}$ and $x_{j}^{H}$. Thus k=2 and the state space indicated in (7) is 16-dimensional.

*Scrambled diagonal* ($D_{4}\times Z_{2}(\rho )$*symmetry*): Rivalry between the images in Fig. 12b leads to the network in Fig. 13b, which has four attributes, two levels, and two types of excitatory coupling, just as in the conventional four-dot experiment. The symmetry group and the maximally fused points are identical to those in the conventional four-dot network.

*Scrambled adjacent* ($D_{2}(\kappa ,\tau )\times Z_{2}(\rho )$): Rivalry between the images in Fig. 12c leads to the network in Fig. 13c. This network has three types of excitatory couplings connecting the nodes in learned patterns. These types are distinguished by the features of equal color and equal distance. The symmetry group of this network is $D_{2}(\kappa ,\tau )\times Z_{2}(\rho )$, where the nonidentity elements in $D_{2}$ are

$$\begin{array}{rcl} \kappa & = & (1\;3)(5\;7)(2\;4)(6\;8),\\ \tau & = & (1\;7)(3\;5)(2\;8)(4\;6),\\ \kappa \tau & = & (1\;5)(2\;6)(3\;7)(4\;8). \end{array}$$

This symmetry group acts transitively on the network nodes; thus all node coordinates are equal in the maximal fusion states as in (8).

*Scrambled unbalanced* ($Z_{2}(\sigma )\times Z_{2}(\rho )$): Rivalry between the images in Fig. 12d leads to the network in Fig. 13d, which has four types of excitatory couplings connecting the nodes in learned patterns. As in the previous network, these types are distinguished by the features of equal color and equal distance. The symmetry group of this network is $Z_{2}(\sigma )\times Z_{2}(\rho )$ where

σ=(15)(26).

The maximal fusion states for this network have the form

$$(\begin{array}{cccc} x & y & x & z\\ x & y & x & z \end{array}).$$

### 5.2 Hopf Bifurcation and Isotypic Components

Suppose that a group *Γ* acts on a vector space *V* with an irreducible subspace *W*. Then an *isotypic component* of that action is the sum of all representations of *Γ* in *V* that are isomorphic to *W*. The theory of equivariant Hopf bifurcations states that such bifurcations occur generically with a center subspace in one of the isotypic components of the symmetry group acting on phase space. Moreover, there can be a Hopf bifurcation type for each isotypic component. See [[21], Chap. XVI] or [[22], Chap. 4]. 

As an example of isotypic components, consider the conventional network with $\Gamma =D_{4}⊕Z_{2}(\rho )$ symmetry and 8*k*-dimensional state space *V* given in (7). It is straightforward to check that

$$V=V_{1}^{+}⊕V_{1}^{-}⊕V_{2}^{-}⊕W,$$

where

$$\begin{array}{rcl} V_{1}^{+} & = & (\begin{array}{cccc} x & x & x & x\\ -x & -x & -x & -x \end{array}),\;\;V_{1}^{-}=(\begin{array}{cccc} x & -x & x & -x\\ -x & x & -x & x \end{array}),\\ V_{2}^{-} & = & (\begin{array}{cccc} x & y & -x & -y\\ -x & -y & x & y \end{array}),\;\;W=(\begin{array}{cccc} x & y & z & u\\ x & y & z & u \end{array}). \end{array}$$

Specifically note that $\dim V_{1}^{+}=\dim V_{1}^{-}=k$, $\dim V_{2}^{-}=2k$, and dimW=4k, and these dimensions add up to dimV=8k. Also note that each summand is invariant under the action of *Γ* and the first three are isotypic components. In fact, we could have decomposed *W* itself into three isotopic components, but we chose not to since the symmetry *ρ* acts trivially on *W*, for reasons that we now explain.

In our theory we consider only those Hopf bifurcations that can lead to non-fusion periodic states. Note that the transposition *ρ* is a symmetry for all of the networks in Fig. 13 and that *ρ* either acts trivially on an isotypic component or as multiplication by −1. In the former case, all bifurcating periodic states have the form

$$X(t)=(\begin{array}{cccc} x_{1}(t) & x_{3}(t) & x_{5}(t) & x_{7}(t)\\ x_{1}(t) & x_{3}(t) & x_{5}(t) & x_{7}(t) \end{array}),$$

all of which are fusion states. Hence, we need only identify those isotypic components for which *ρ* acts as multiplication by −1. These bifurcations all lead to *T*-periodic solutions of the form

$$X(t)=(\begin{array}{cccc} x_{1}(t) & x_{3}(t) & x_{5}(t) & x_{7}(t)\\ x_{1}(t+\frac{T}{2}) & x_{3}(t+\frac{T}{2}) & x_{5}(t+\frac{T}{2}) & x_{7}(t+\frac{T}{2}) \end{array}).$$

The isotypic components where *ρ* acts as minus the identity are listed in Table 1 for each of the four networks. There are three such isotypic components for each of conventional and scrambled diagonal networks. The first two ($V_{1}^{+}$ and $V_{1}^{-}$) lead to the percepts in Figs. 12a and 12b; however, they interchange the learned and derived percepts in these two experiments. 

**Table 1:** Isotypic components corresponding to representations where *ρ* acts as −1 in the four-dot networks

Experiment	Symmetries	*V*	Subspace	Kernel
Conventional 4-dot	$D_{4}⊕Z_{2}(\rho )$	$V_{1}^{+}$	$\left(\begin{array}{cccc} x & x & x & x\\ -x & -x & -x & -x \end{array}\right)$	$D_{4}$
		$V_{1}^{-}$	$\left(\begin{array}{cccc} x & -x & x & -x\\ -x & x & -x & x \end{array}\right)$	
		$V_{2}^{-}$	$\left(\begin{array}{cccc} x & y & -x & -y\\ -x & -y & x & y \end{array}\right)$	**1**
Scrambled diagonal	$D_{4}⊕Z_{2}(\rho )$	$V_{1}^{+}$	$\left(\begin{array}{cccc} x & -x & x & -x\\ -x & x & -x & x \end{array}\right)$	$D_{4}$
		$V_{1}^{-}$	$\left(\begin{array}{cccc} x & x & x & x\\ -x & -x & -x & -x \end{array}\right)$	
		$V_{2}^{-}$	$\left(\begin{array}{cccc} x & y & -x & -y\\ -x & -y & x & y \end{array}\right)$	**1**
Scrambled adjacent	$D_{2}(\kappa ,\tau )⊕Z_{2}(\rho )$	$V_{1}$	$\left(\begin{array}{cccc} x & x & x & x\\ -x & -x & -x & -x \end{array}\right)$	$D_{2}$
		$V_{2}$	$\left(\begin{array}{cccc} x & x & -x & -x\\ -x & -x & x & x \end{array}\right)$	$Z_{2}(\kappa )$
		$V_{3}$	$\left(\begin{array}{cccc} x & -x & -x & x\\ -x & x & x & -x \end{array}\right)$	$Z_{2}(\tau )$
		$V_{4}$	$\left(\begin{array}{cccc} x & -x & x & -x\\ -x & x & -x & x \end{array}\right)$	$Z_{2}(\kappa \tau )$
Scrambled unbalanced	$Z_{2}(\sigma )⊕Z_{2}(\rho )$	$V_{1}$	$\left(\begin{array}{cccc} x & y & x & z\\ -x & -y & -x & -z \end{array}\right)$	$Z_{2}(\sigma )$
		$V_{2}$	$\left(\begin{array}{cccc} x & 0 & -x & 0\\ -x & 0 & x & 0 \end{array}\right)$	$Z_{2}(\sigma \rho )$

**Fig. 14:**
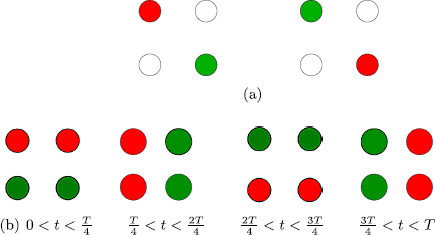
New patterns of oscillation predicted by a $V_{2}^{-}$ Hopf bifurcation for the conventional *four-dot* experiment: fusion (**a**) and rotating wave (**b**)

The third bifurcation type $V_{2}^{-}$ leads to three types of solutions: rivalry with adjacent dots of the same color such as in Fig. 12c, a fusion state as in Fig. 14a, and most interestingly a rotating wave state as in Fig. 14b. More precisely, the three solution types have the form

$$\begin{array}{cc} \left(\begin{array}{cccc} a_{0} & a_{0} & a_{1/2} & a_{1/2}\\ a_{1/2} & a_{1/2} & a_{0} & a_{0} \end{array}\right) & \left(\begin{array}{cccc} a_{0} & b_{0} & a_{1/2} & b_{0}\\ a_{1/2} & b_{0} & a_{0} & b_{0} \end{array}\right)\\ \text{adjacent dots rivalry} & \text{fusion}\\ (\begin{array}{cccc} a_{0} & a_{1/4} & a_{2/4} & a_{3/4}\\ a_{1/2} & a_{3/4} & a_{0} & a_{1/4} \end{array}),\\ \text{rotating wave} \end{array}$$

where *b* is twice the frequency of *a*. See the discussion of Hopf bifurcation with $D_{4}$ symmetry in [21]. We can describe the percepts associated with these bifurcations more simply. The only non-fusion states are scrambled adjacent states, where the two red dots can be in any of the four orientations. 

*Scrambled adjacent network*: The isotypic components where *ρ* acts nontrivially are listed in Table 1. The alternations predicted by each of the associated Hopf bifurcations are as follows: $V_{1}$ yields Fig. 12a; $V_{2}$ yields Fig. 12c; $V_{3}$ yields Fig. 12c rotated by 90^∘^, and $V_{4}$ yields Fig. 12b.

*Scrambled unbalanced*: There are two possible isotypic components where *ρ* acts nontrivially and either *σ* acts trivially or *σ* acts nontrivially. See Table 1. The solutions corresponding to $V_{2}$ have $x_{3}(t)$ and $x_{7}(t)$ oscillating at double frequency. However, these states are fusion states, as shown in Fig. 14b. The exact temporal ordering of percepts corresponding to $V_{1}$ solutions is complicated to explain. However, the percepts themselves are straightforward. The colors of dots UL and LR are always the same and alternate between red and green. The colors of the dots LL and UR also alternate between red and green, but the times of the alternations can be different. The result is that all eight four-dot percepts where the UL and LR dots have the same color can appear in a $V_{1}$ solution type.

#### 5.2.1 Comparison with the Four-Dot Experiment Analysis in [13]

The analysis of the *conventional* and *scrambled diagonal* 4-dot experiments presented here is related to, but definitely different from, the one that we gave in [13]. In [13], we followed Wilson [7] by assuming that all excitatory couplings between attribute nodes in the learned patterns were the same. This is a reasonable hypothesis but it leads to the conclusion that the symmetry groups of both 4-dot experiments are $S_{4}\times Z_{2}(\rho )$; that is, permuting the attribute columns arbitrarily is permitted. Fusion-breaking Hopf bifurcation with $S_{4}$ symmetry does not lead easily to the existence of alternation between the pure-color images in the scrambled 4-dot experiment. However, in [13], when we added a second type of coupling between nodes representing the same color, which we called *lateral coupling* by analogy with the known architecture of the primary visual cortex, then the symmetry group in the scrambled 4-dot experiment is (the desired) $D_{4}\times Z_{2}(\rho )$. We now see that this change in symmetry is better achieved by assuming that the excitatory couplings in our networks are the same only when certain features are preserved, which is consistent with Hebbian learning.

## 6 Three Colored-Dot Experiments

**Fig. 15:**
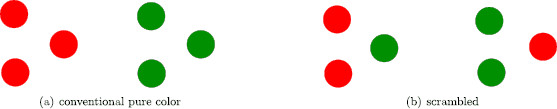
Learned images for two-color *three-dot* experiments

Experiments using three rather than four colored dots should be simpler to perform. Moreover, there are only two types of such experiments: the conventional experiment where the left eye is shown three red dots and the right eye three green dots (Fig. 15a), and the scrambled experiment where the left eye is shown two red dots and one green dot and the right eye is shown the complementary pattern (Fig. 15b). Our models for these two experiments predict different percepts, as we now explain. The pure-color three-dot experiment is analogous to the pure-color four-dot experiment and the scrambled three-dot experiment is analogous to the scrambled unbalanced four-dot experiment. 

**Fig. 16:**
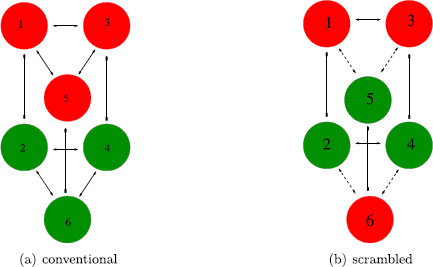
Rivalry networks for two-color *three-dot* experiments. *Solid lines with arrowheads* indicate excitatory coupling between *dots of the same color*, and *dashed lines with arrowheads* indicate excitatory coupling between *dots of different colors*

The networks for the two three-dot experiments are shown in Fig. 16. The three attributes in these experiments are the colors of the dots at the three geometric positions upper left (UL), lower left (LL), and right (R). The two levels for each attribute are the colors red and green. 

There is only one type of excitatory coupling in the pure-color experiment Fig. 16a and its symmetry group is $D_{3}\times Z_{2}(\rho )$. Note that $D_{3}$ permutes the three attribute columns arbitrarily and

ρ=(12)(34)(56).

There are two types of excitatory couplings in the scrambled experiment Fig. 16b suggested by the color feature. The symmetry group of this network is $Z_{2}(\kappa )\times Z_{2}(\rho )$ where

κ=(13)(24).

### 6.1 Hopf Bifurcation and Isotypic Components

As in the four-dot networks we consider only those Hopf bifurcations from a maximal fusion state that can lead to non-fusion periodic states. The states are denoted

$$X=(\begin{array}{ccc} x_{1} & x_{3} & x_{5}\\ x_{2} & x_{4} & x_{6} \end{array}).$$

In the conventional experiment the group acts transitively and the nodes 1–6 are equal in the maximal fusion state, whereas in the scrambled experiment the group does not act transitively and a maximal fusion state has the form

$$(\begin{array}{ccc} a & a & b\\ a & a & b \end{array}).$$

As in the four-dot experiments the transposition *ρ* either acts trivially on an isotypic component or as multiplication by −1. Bifurcations in the former case lead to fusion states. Hence, we need only identify those isotypic components for which *ρ* acts as multiplication by −1 and these bifurcations all lead to *T*-periodic solutions of the form

$$X(t)=(\begin{array}{ccc} x_{1}(t) & x_{3}(t) & x_{5}(t)\\ x_{1}(t+\frac{T}{2}) & x_{3}(t+\frac{T}{2}) & x_{5}(t+\frac{T}{2}) \end{array}).$$

The conventional and scrambled networks each have two isotypic components where *ρ* acts as minus the identity. These components are listed in Table 2. 

**Table 2:** Isotypic components corresponding to representations where *ρ* acts as −1 in the three-dot networks

Experiment	Symmetries	*V*	Subspace	Kernel
Conventional 3-dot	$D_{3}⊕Z_{2}(\rho )$	$V_{1}$	$\left(\begin{array}{ccc} x & x & x\\ -x & -x & -x \end{array}\right)$	$D_{3}$
		$V_{2}$	$\left(\begin{array}{ccc} x & y & -x-y\\ -x & -y & x+y \end{array}\right)$	**1**
Scrambled unbalanced	$Z_{2}(\kappa )⊕Z_{2}(\rho )$	$V_{1}$	$\left(\begin{array}{ccc} x & x & y\\ -x & -x & -y \end{array}\right)$	$Z_{2}(\kappa )$
		$V_{2}$	$\left(\begin{array}{ccc} x & -x & 0\\ -x & x & 0 \end{array}\right)$	$Z_{2}(\kappa \rho )$

### 6.2 Three-Dot Conventional Experiment

There are two types of fusion-breaking Hopf bifurcations corresponding to the two irreducible representations of $D_{3}\times Z_{2}(\rho )$ with *ρ* acting as multiplication by −1. The 1D trivial irreducible representation $V_{1}$ of $D_{3}$ leads to standard learned pattern rivalry between the pure-color images in Fig. 15a. That is, the corresponding periodic solutions have the form

(9) $$(\begin{array}{ccc} x_{0} & x_{0} & x_{0}\\ x_{1/2} & x_{1/2} & x_{1/2} \end{array}).$$

The 2D irreducible representation $V_{2}$ leads to three solution types: rotating waves, two nodes in phase with the third node phase-shifted from the other two, and two nodes out of phase with the third node fused. We produce pictures representative of the periodic solutions obtained via Hopf bifurcation by using the functions

(10) $$\begin{array}{rcl} x_{\theta }(t) & = & \cos\left(2\pi (t+\theta )\right),\\ y_{\theta }(t) & = & \cos\left(2\pi (t+\theta -0.2)\right),\\ z_{\theta }(t) & = & \cos\left(4\pi (t+\theta -0.2)\right). \end{array}$$

• Rotating wave:

(11) $$(\begin{array}{ccc} x_{0} & x_{2/6} & x_{4/6}\\ x_{3/6} & x_{5/6} & x_{1/6} \end{array}).$$

• Two dots in-phase:

(12) $$(\begin{array}{ccc} x_{0} & x_{0} & y_{0}\\ x_{1/2} & x_{1/2} & y_{1/2} \end{array}).$$

**Fig. 17:**
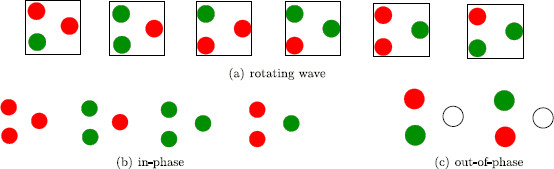
Percepts obtained from a symmetry-breaking $V_{2}$ Hopf bifurcation

**Fig. 18:**
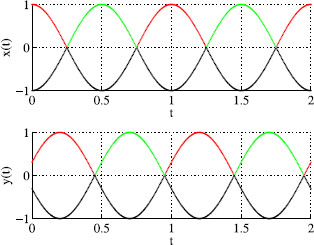
Example wave forms x(t) and y(t) produced using (10)

Symmetry does not force x(t) and y(t) to have identical phase-shifted wave forms. Thus, typically, there will be a nonzero phase shift in color switching between nodes 1 and 3 (see Fig. 17b). More precisely, observe in Fig. 18 that the times when color alternation occurs need not be the same in the two waves. 

• Two dots out of phase:

(13) $$(\begin{array}{ccc} x_{0} & x_{1/2} & z_{0}\\ x_{1/2} & x_{0} & z_{1/2} \end{array}).$$

Since *z* has double frequency, $z_{0}=z_{1/2}$; so this final state is a partial fusion state as in Fig. 17c.

### 6.3 Three-Dot Scrambled Experiment

In the two Hopf bifurcations from a maximal fusion state where *ρ* acts nontrivially (as multiplication by −1), *κ* acts trivially in one and nontrivially in the other. These bifurcations lead to rivalry or partial rivalry. 

• *κ* acts trivially:

(14) $$(\begin{array}{ccc} x_{0} & x_{0} & y_{0}\\ x_{1/2} & x_{1/2} & y_{1/2} \end{array}).$$

This solution is similar to “rivalry” between the two learned patterns, but it generically will have derived patterns in between. The times when the like-colored dots switch are different due to the phase shift between $x_{0}$ and $y_{0}$. This time-periodic solution is the same as the one in Fig. 17b.

• *κ* acts nontrivially:

(15) $$(\begin{array}{ccc} x_{0} & x_{1/2} & z_{0}\\ x_{1/2} & x_{0} & z_{1/2} \end{array}).$$

In this solution, the third node is fused, and the solution is the same as the one in Fig. 17c.

In short, the only expected non-fusion percepts in the unbalanced scrambled case are the percepts in Fig. 17b. In particular, we do not expect simple alternation between the two learned patterns.

## 7 Summary of Colored Dot Experiments and Predictions

There are four different two-color four-dot experiments where the pictures shown to the right and left eyes have red and green dots interchanged; see Fig. 12. There are just two such two-color three-dot experiments shown in Fig. 15. Our theoretical analysis leads to interesting differences between these experiments. 

(1) Our theory suggests that the expected percepts for the first two four-dot rivalry experiments (conventional pure-color Fig. 12a and scrambled diagonal Fig. 12b) are identical.

(2) Both conventional and scrambled diagonal four-dot experiments admit the possibility of a discrete rotating wave (Fig. 14c), whereas the other two four-dot experiments do not.

(3) Suppose the single red dot in the four-dot scrambled unbalanced experiment (Fig. 12d) occurs in the UR position. Then all percepts should have the same color in the UL and LR positions. The scrambled unbalanced experiment is the only one of the four-dot experiments where simple alternation between the two learned patterns is unexpected.

(4) The conventional pure-color three-dot experiment (Fig. 15a) leads to simple alternation between (learned pattern) pure-color percepts, but it does not lead to simple alternation between mixed-color percepts. It can also lead to discrete rotating waves.

(5) The unbalanced scrambled three-dot experiment (Fig. 15b) is not predicted to lead to alternation between either pure-color or mixed-color percepts (the learned patterns). This experiment is also not expected to lead to rotating waves.

(6) The unbalanced scrambled three-dot experiment is expected to lead to mixed-color percept alternation occurring with pure-color percepts in between (see Fig. 17b). In this alternation the color of dots UL and LL should be the same. This experiment could also lead to percepts where UL and LL alternate with opposite colors and R has an indefinite color (fused state).

## 8 Definition of a Rivalry Network

The possibility that there may be several types of attributes (see the network for the Shevell et al. [17] experiment in Fig. 8) and several types of excitatory couplings (see the networks for the four-dot experiments in Fig. 12 as well as the network in Fig. 8) are the two principal differences between Wilson networks and rivalry networks. These generalizations seem to be necessary to explain the percepts found in the experiments that we have described. The need for additional coupling types was discussed in [13], where we introduced lateral couplings. Here we describe a simpler way of introducing multiple excitatory couplings, based on Hebbian learning, which we formalize through the definition of a feature below. 

Following [19] a coupled cell network consists of a collection of nodes or cells , a collection of arrows or edges ℰ, equivalence classes of cells, and equivalence classes of edges.

**Definition 3** A *Wilson network* is a coupled cell network satisfying the following: 

(a) The nodes are partitioned into a disjoint union of *mattribute columns*; that is,

$$C=A_{1}\cup \cdots \cup A_{m},$$

where all nodes in an attribute column are cell equivalent. A node in an attribute column is a *level* of that attribute.

(b) A *pattern* is a choice of one node from each attribute. There is a distinguished collection of *learned patterns*; all other patterns are called *derived*.

(c) There are two types of arrows: *inhibitory* and *excitatory*. 

• Each pair of nodes in the same attribute column is connected by a single inhibitory arrow.

• Each pair of nodes in the same learned pattern is connected by a single excitatory arrow. (If a pair of nodes is in *p* patterns, then that pair will be connected by *p* arrows.)

 Inhibitory arrows and excitatory arrows are not arrow equivalent.

*Wilson network discussion*: An attribute could be the color of a dot and the levels the possible colors. In binocular rivalry experiments, the network has two different learned patterns. In the language of [19], Wilson considers networks where all nodes are cell equivalent, all inhibitory arrows are arrow equivalent, and all excitatory arrows are arrow equivalent. Rivalry networks need not have these properties. 

**Definition 4** A *rivalry network* is a Wilson network such that: 

(d) Attribute columns partition into *attribute equivalent columns* such that 

• Nodes are cell equivalent if and only if they are in attribute equivalent columns.

• Inhibitory arrows are arrow equivalent if and only if they are associated with equivalent attributes.

• Attribute equivalent columns have the same number of levels.

(e) A *feature* is defined on pairs of nodes in a subset of attribute columns. Two excitatory arrows *i* and *j* are arrow equivalent if and only if the features associated to the head and tail nodes of *i* are identical to the features associated with the head and tail nodes of *j*.

*Rivalry network discussion*: In Fig. 8 there are two types of attribute columns: those associated with colors and those associated with grating directions. Partitioning attribute columns into types admits the possibility of cell inequivalent nodes and arrow inequivalent inhibitory arrows. Features admit the possibility of arrow inequivalent excitatory arrows. Here are two examples of features: 

(1) The *level feature* identifies pairs of nodes in two attribute equivalent columns as either having the same level or having a different level. For example, suppose $A_{k}$ is the attribute column defining the color of dot *k*. Then the level feature identifies pairs of levels in columns $A_{1}$ and $A_{2}$ as either having the same color or having different colors.

(2) The color dot experiments have a *distance feature* that assigns to any pair of nodes the geometric distance between the associated dots. Thus two excitatory arrows can be equivalent only if their head and tail nodes correspond to equidistant dots.

 We have presumed that Hebbian learning strengthens excitatory connections between nodes with the same feature, so that in the end the strength of excitatory connections between pairs of nodes can be identical only if those pairs have the same set of features.

## 9 Conclusion

It requires some thought to define a rivalry network for any particular rivalry experiment. We have shown here that reasonable descriptions of rivalry networks for a variety of experiments lead (via the use of fusion-breaking Hopf bifurcations) to the prediction of the percepts that are perceived in these experiments. To our knowledge, the form of percepts have not previously been predicted.

Periodic states that are near Hopf bifurcation can exhibit alternation only if that Hopf bifurcation is from a fusion state. If not, the activity of one oscillating node would be bigger than the activity of other oscillating nodes for all time and alternation would not occur. It follows that alternation obtained from small amplitude periodic solutions can only occur robustly in a model network when fusion states are structurally stable in that network. For this reason, we propose that the structure inherent in rivalry networks that leads to fusion states is required to describe alternation.

Several of the rivalry experiments we have discussed exhibit interocular grouping, that is, components of the left eye and right eye images are combined to achieve a single coherent percept [24,25]. Such groupings occur when learned images can be naturally subdivided and reassembled in other forms. The subdivision process seems to be captured by the attributes and their types (which define node types) and the features (which define excitatory coupling types), as has been shown in the experiments considered here. Together the attributes and features define the rivalry networks and their symmetries. The mathematics of symmetry-breaking Hopf bifurcation leads to an enumeration of the likely ways that the subdivisions can be reassembled, that is, to a set of interocular groupings associated with percepts. 

The choice of a network for a rivalry experiment is not unique. For example, in Sect. 2 we choose to model the monkey-text experiment with a two-attribute network. A possible alternative network is one with six attributes, corresponding to the six distinct areas shown in Fig. 5a (without grouping them into blue and white regions). This leads to a 12-node network as a model for this experiment, rather than the 4-node network of Fig. 5b. However, there is a relationship between these two networks. The larger network has a *quotient network* on a flow-invariant subspace that is isomorphic to the network in Fig. 5b (see [20] and Sect. 5 of [13]). Thus, the bifurcations in the smaller model are a subset of the bifurcations in the larger model. The solution types discussed in Sect. 2 for the smaller model will also appear in the model with a more refined geometry, however, the larger model can also exhibit other more complicated dynamics not found in the simpler model. In summary, choosing an appropriate network for a given experiment involves trade-offs and is an iterative process.

We focused here on predicting the form of percepts, which depends on network structure but not on specific model equations. A combined study of specific equations on specific networks, analogous to the work of [3,4] on two-unit models, can be contemplated. 

## Competing Interests

The authors declare that they have no competing interests.

## Authors’ Contributions

Both authors performed research, drafted the manuscript, and read and approved the final manuscript.

## References

[B1] Blake R Logothetis NK Visual competition Nat Rev Neurosci 2002 4 1 11 10.1038/nrn701 11823801

[B2] Laing C Chow CC A spiking neuron model for binocular rivalry J Comput Neurosci 2002 4 39 53 10.1023/A:1014942129705 11932559

[B3] Shpiro A Curtu R Rinzel J Rubin N Dynamical characteristics common to neuronal competition models J Neurophysiol 2007 4 462 473 17065254 10.1152/jn.00604.2006 PMC2702527

[B4] Curtu R Shpiro A Rubin N Rinzel J Mechanisms for frequency control in neuronal competition models SIAM J Appl Dyn Syst 2008 4 609 649 10.1137/070705842 20953287 PMC2954747

[B5] Kilpatrick ZP Bressloff PC Binocular rivalry in a competitive neural network with synaptic depression SIAM J Appl Dyn Syst 2010 4 1303 1347 10.1137/100788872

[B6] Seely J Chow CC The role of mutual inhibition in binocular rivalry J Neurophysiol 2011 4 2136 2150 10.1152/jn.00228.2011 21775721 PMC3296268

[B7] Wilson HR Jenkins M, Harris L Requirements for conscious visual processing Cortical Mechanisms of Vision 2009 Cambridge University Press, Cambridge 399 417

[B8] Wilson HR Miller SM Binocular rivalry: cooperation, competition, and decisions The Constitution of Visual Consciousness: Lessons from Binocular Rivalry 2013 Benjamins, Amsterdam 281 304

[B9] Tononi G Edelman GM Consciousness and complexity Science 1998 4 1846 1851 9836628 10.1126/science.282.5395.1846

[B10] Dehaene S Kerszberg C Changeux JP A neuronal model of a global workspace in effortful cognitive tasks Proc Natl Acad Sci USA 1998 4 14529 14534 10.1073/pnas.95.24.14529 9826734 PMC24407

[B11] Dehaene S Sergent C Changeux JP A neuronal network model linking subjective reports and objective physiological data during conscious perception Proc Natl Acad Sci USA 2003 4 8520 8525 10.1073/pnas.1332574100 12829797 PMC166261

[B12] Crick F Koch C A framework for consciousness Nat Neurosci 2003 4 119 126 10.1038/nn0203-119 12555104

[B13] Diekman C Golubitsky M Wang Y Derived patterns in binocular rivalry networks J Math Neurosci 2013 10.1186/2190-8567-3-6 PMC3698087 23657206

[B14] Kovács I Papathomas TV Yang M Fehér A When the brain changes its mind: interocular grouping during binocular rivalry Proc Natl Acad Sci USA 1996 4 15508 15511 10.1073/pnas.93.26.15508 8986842 PMC26435

[B15] Suzuki S Grabowecky M Evidence for perceptual “trapping” and adaptation in multistable binocular rivalry Neuron 2002 4 143 157 10.1016/S0896-6273(02)00934-0 12367513

[B16] Hong SW Shevell SK Resolution of binocular rivalry: perceptual misbinding of color Vis Neurosci 2006 4 561 566 16961996 10.1017/S0952523806233145

[B17] Shevell SK St Clair R Hong SW Misbinding of color to form in afterimages Vis Neurosci 2008 4 355 360 18321397 10.1017/S0952523808080085

[B18] Diekman C Golubitsky M McMillen T Wang Y Reduction and dynamics of a generalized rivalry network with two learned patterns SIAM J Appl Dyn Syst 2012 4 1270 1309 10.1137/110858392

[B19] Golubitsky M Stewart I Török A Patterns of synchrony in coupled cell networks with multiple arrows SIAM J Appl Dyn Syst 2005 4 78 100 10.1137/040612634

[B20] Golubitsky M Stewart I Nonlinear dynamics of networks: the groupoid formalism Bull Am Math Soc 2006 4 305 364 10.1090/S0273-0979-06-01108-6

[B21] Golubitsky M Stewart I Schaeffer DG Singularities and Groups in Bifurcation Theory: Volume II Applied Mathematical Sciences 69 1988 Springer, New York

[B22] Golubitsky M Stewart I The Symmetry Perspective Progress in Mathematics 200 2002 Birkhäuser, Basel

[B23] Tong F Meng M Blake R Neural bases of binocular rivalry Trends Cogn Sci 2006 4 502 511 10.1016/j.tics.2006.09.003 16997612

[B24] Lee S Blake R A fresh look at interocular grouping during binocular rivalry Vis Res 2004 4 983 991 10.1016/j.visres.2003.12.007 15031091

[B25] Papathomas TV Kovács I Conway T Alais D, Blake R Interocular grouping in binocular rivalry: basic attributes and combinations Binocular Rivalry 2005 MIT Press, Cambridge 155 168

